# The Influence of Snowmobile Trails on Coyote Movements during Winter in High-Elevation Landscapes

**DOI:** 10.1371/journal.pone.0082862

**Published:** 2013-12-18

**Authors:** Eric M. Gese, Jennifer L. B. Dowd, Lise M. Aubry

**Affiliations:** 1 United States Department of Agriculture, Wildlife Services, National Wildlife Research Center, Department of Wildland Resources, Utah State University, Logan, Utah, United States of America; 2 Department of Wildland Resources, Utah State University, Logan, Utah, United States of America; Bangor University, United Kingdom

## Abstract

Competition between sympatric carnivores has long been of interest to ecologists. Increased understanding of these interactions can be useful for conservation planning. Increased snowmobile traffic on public lands and in habitats used by Canada lynx (*Lynx canadensis*) remains controversial due to the concern of coyote (*Canis latrans*) use of snowmobile trails and potential competition with lynx. Determining the variables influencing coyote use of snowmobile trails has been a priority for managers attempting to conserve lynx and their critical habitat. During 2 winters in northwest Wyoming, we backtracked coyotes for 265 km to determine how varying snow characteristics influenced coyote movements; 278 km of random backtracking was conducted simultaneously for comparison. Despite deep snow (>1 m deep), radio-collared coyotes persisted at high elevations (>2,500 m) year-round. All coyotes used snowmobile trails for some portion of their travel. Coyotes used snowmobile trails for 35% of their travel distance (random: 13%) for a mean distance of 149 m (random: 59 m). Coyote use of snowmobile trails increased as snow depth and penetrability off trails increased. Essentially, snow characteristics were most influential on how much time coyotes spent on snowmobile trails. In the early months of winter, snow depth was low, yet the snow column remained dry and the coyotes traveled off trails. As winter progressed and snow depth increased and snow penetrability increased, coyotes spent more travel distance on snowmobile trails. As spring approached, the snow depth remained high but penetrability decreased, hence coyotes traveled less on snowmobile trails because the snow column off trail was more supportive. Additionally, coyotes traveled closer to snowmobile trails than randomly expected and selected shallower snow when traveling off trails. Coyotes also preferred using snowmobile trails to access ungulate kills. Snow compaction from winter recreation influenced coyote movements within an area containing lynx and designated lynx habitat.

## Introduction

Intraspecific competition between sympatric carnivores has long been of interest to ecologists and managers. Understanding the interactions and fundamental relationships between competing species can lead to more informed management and conservation decisions. Coyotes (*Canis latrans*) and Canada lynx (*Lynx canadensis*) are sympatric carnivores in many areas of North America. Conservation and management activities for Canada lynx in the contiguous United States have increased to enhance species recovery and protect critical habitats. Since their listing in 2000 [1], determining appropriate management approaches to minimize adverse impacts and maximize species recovery is essential for many land agencies managing lynx habitat [2]. Concerns regarding the relationship between snowmobile activity and coyote presence within winter habitats used by lynx remain a focal point for many management agencies. Conflicting pieces of information suggest varying degrees of coyote dependence on snowmobile trails [3]–[4], and therefore the potential for varying impacts of coyotes on local lynx populations. We hypothesize that the regional differences in snow depth and supportiveness, terrain, recreation use, lynx density, available food, suitable habitat, and/or species dynamics may account for this variation in the dependence of coyotes using trails compacted by snowmobiles [3]–[4].

Coyotes are one of the most successful generalist predators in North America and are highly adaptive to human-modified environments. In regions where seasonal activity is dictated by winter climates, coyotes alter their behaviors to negate the impacts of deep snow by using areas and habitats where snow is shallower and more supportive [4]–[5]. Due to their high foot-load to body-mass, coyotes have a greater sinking depth than lynx, thereby making travel and hunting in deep snow terrains more energetically expensive [6]. Lynx have specially adapted feet resulting in a lower foot-load to body-mass, giving them a competitive advantage over coyotes during winter [7]–[9]. Therefore, ecologists have hypothesized that where coyotes and lynx inhabit the same geographical areas, the two species may occupy separate niches seasonally due to fluctuations in snow characteristics, with coyote's primarily occurring in lower elevations with more supportive snow during winter and lynx occurring in higher elevations with deeper snow [5]. However, this hypothesis remains largely untested. Increased winter recreation creates an increase of compacted snow surfaces, thereby providing an opportunity for coyotes to exploit deep snow conditions and utilize resources year round. In the Intermountain West, coyotes have been documented using snowmobile trails to travel, hunt and persist in otherwise inaccessible winter terrain [3]. Researchers suggested the continued use of snowmobiles may result in consistent compacted trails within lynx conservation areas which may have detrimental impacts to local lynx populations in the Intermountain West [3].

The growing popularity of snowmobiles, combined with recent technological advances (lighter and more powerful snowmobiles), has enabled greater access to backcountry terrain, expansion of trail grooming, and an increase in off-trail use by winter recreationists. In light of this, management has focused on determining if snowmobile use has the potential to influence ecosystem dynamics. Studies suggest increased competition between coyotes and lynx resulting from snow compaction would most likely occur during the fall and winter [4], [10]–[11] when coyotes use snow-compacted paths to travel and hunt [3], [7], [12]. Understanding how coyote behaviors are influenced by winter recreation (particularly their use of snowmobile trails within habitats used by lynx) is necessary for understanding how lynx populations might be impacted by management plans in critical lynx habitat. The objective of this study was to quantify the influence of snow compaction created by snowmobiles on coyote winter movements in deep snow terrain, with a comparison to the only study [4] using similar field collection methodologies. This comparison will be useful to inform land management agencies that regional differences in winter precipitation regimes (i.e., snow depth, snow compaction) may lead to different interactions among coyotes, lynx, and snowmobiles.

## Methods

### Ethics Statement

Fieldwork was approved and sanctioned by the United States Department of Agriculture's National Wildlife Research Center and the United States Forest Service. Permission to access land in the Bridger-Teton National Forest was obtained from the United States Forest Service.

Capture and handling protocols were reviewed and approved by the Institutional Animal Care and Use Committees (IACUC) at the United States Department of Agriculture's National Wildlife Research Center (QA-1389) and Utah State University (#1294). No permit to capture and handle coyotes was required by the Wyoming Game and Fish Department.

### Study Area

We conducted this study on the east and west sides of Togwotee Pass in northwestern Wyoming. The 512-km^2^ study area was composed of the Bridger-Teton and Shoshone National Forests, plus privately owned ranches. Elevations ranged from 1,800 m to >3,600 m. The area was characterized by short, cool summers (mean temperature of 12°C) and long winters (mean temperature of −8°C). Precipitation occurred mostly as snow; cumulative monthly snow depth for the winter study season (December-April) averaged 226.6, 149.4, and 228.9 cm during 2006, 2007, and 2008, respectively [13]. Snowmobiling was extensive during winter with riders accessing both groomed trails and areas for off-trail riding once snow conditions permitted (October through May). Grooming of trails began in December with trails maintained through April 1 depending on snowfall. Wyoming's Continental Divide Snowmobile Trail was considered one of the top trail systems in the west [14].

Habitats varied between the east and west sides of the pass, with the eastern side classified as dry and the western side as wet. Plant communities included cottonwood (*Populus angustifolia*) riparian zones, interspersed with sagebrush (*Artemisia* spp.) uplands and willow (*Salix spp*.) -wetland communities at lower elevations. At intermediate elevations, aspen (*Populus tremuloides*), Douglas fir (*Pseudotsuga menziesii*), and lodgepole pine (*Pinus contorta*) were the dominant species. Whitebark pine (*Pinus albicaulis)*, spruce (*Picea engelmannii*), and sub-alpine fir (*Abies lasiocarpa*) were the primary tree species at higher elevations.

The study area has a diverse assemblage of predators. Although wolves were extirpated from Wyoming by the 1930's, they have since re-established as a result of the 1995 re-introduction efforts in Yellowstone National Park. Other carnivores aside from coyotes and lynx included cougar (*Puma concolor*), wolverine (*Gulo gulo*), grizzly bear (*Ursus arctos*), black bear (*U. americanus*), bobcat (*L. rufus*), red fox (*Vulpes vulpes*), and pine marten (*Martes americana*). Ungulate species in the area included elk (*Cervus elaphus*), moose (*Alces alces*), bison (*Bison bison*), bighorn sheep (*Ovis canadensis*), mule deer (*Odocoileus hemionus*), and white-tailed deer (*O. virginianus*). Pronghorn antelope (*Antilocapra americana*) were in the area only during the summer. Potential prey for coyotes and lynx included snowshoe hare (*Lepus americanus*), red squirrel (*Tamiansciurus hudsonicus*), Uinta ground squirrel (*Spermophilus armatus*), black-tailed jackrabbit (*Lepus californicus*), cottontail rabbit (*Sylvilagus spp*.), ruffed grouse (*Bonasa umbellus*), blue grouse (*Dendragapus obscurus*), northern flying squirrel (*Glaucomys sabrinus*), deer mouse (*Peromyscus maniculatus*), voles (*Microtus spp*.), gophers (*Thomomys spp*.), and various cricetid species.

### Animal Capture and Backtracking

We captured coyotes across the study area in the summer and fall using padded-jaw leg-hold traps with attached tranquilizer tabs. We also captured coyotes during winter by placing road-kill deer and elk carcasses in open meadows around the study area and using snowmobiles with nets, or net-gunning from a helicopter [15]. Coyotes were radio-collared with a very high frequency (VHF) transmitter and released at the capture site; animals were handled without immobilizing drugs. Radio-collared animals were relocated throughout the year using conventional radio-telemetry techniques (homing in or triangulation) to determine year-round territory occupancy, survival, and residency status.

We backtracked radio-collared coyotes during the winters of 2006–2007 and 2007–2008 following methods developed at Seeley Lake, Montana [4], to quantify the influence of snow compaction on coyote movements in an area where lynx, coyotes, and snowmobiles occurred, and allowed for comparison to results from studies in geographically separate regions [4]. In an effort to determine if various snow column characteristics, with particular emphasis on differences in snow supportiveness, would influence the dependence of coyotes on snowmobile trails for movement, we sampled individuals residing on the east, west, and continental divide of Togwotee Pass. We used data collected during the backtracking of individuals to determine the variance from random expectation of the distance a coyote would travel on a snowmobile trail (dependent variable) and the influence of various environmental variables, including the rate of prey and predator track encounters, snow depth, snow penetrability, and the distance a coyote traveled off of the nearest snowmobile trail.

**Figure 1:**
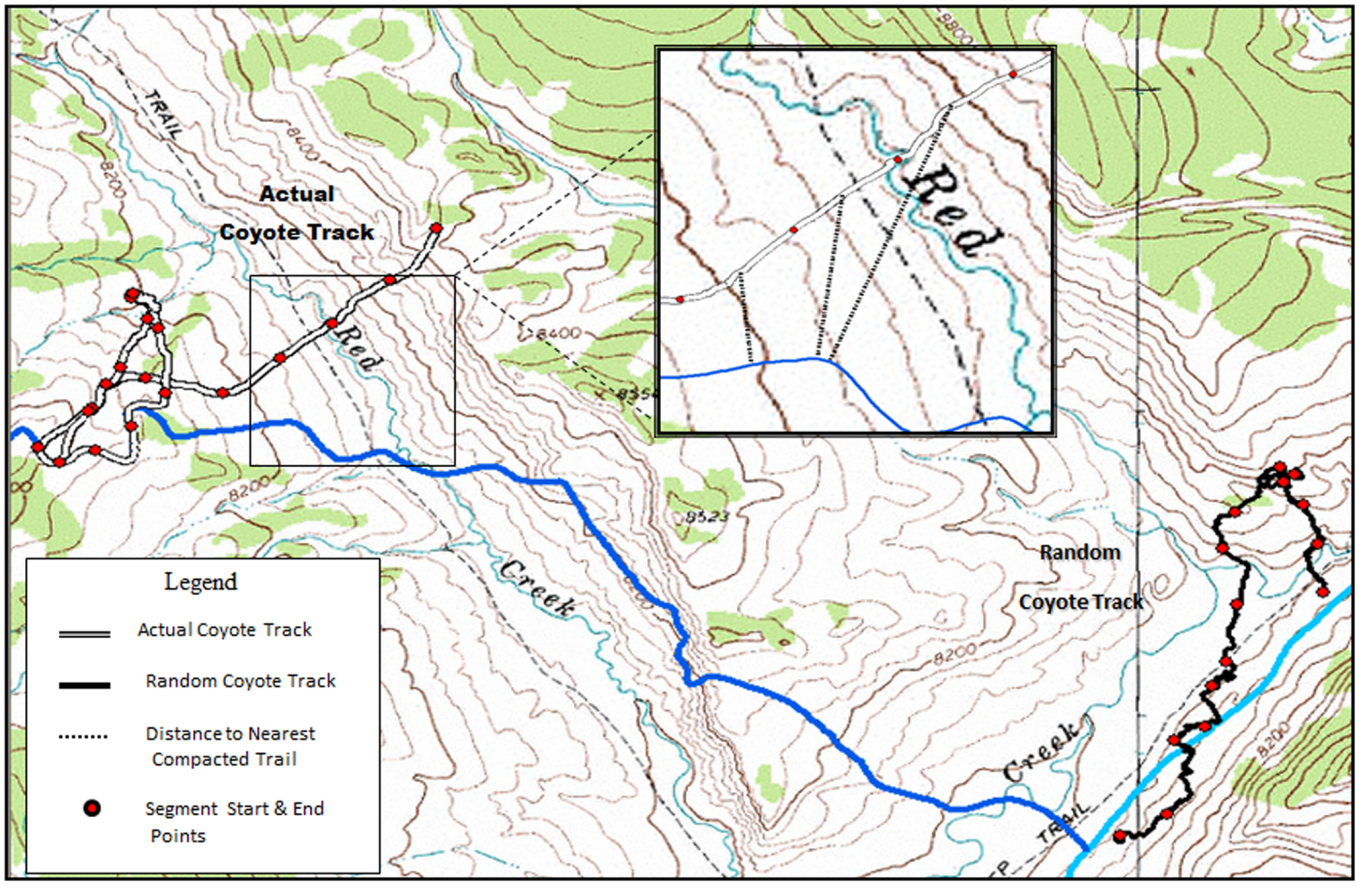
Comparison of an actual and random coyote track documented on 15 February 2008, Togwotee Pass, Wyoming. Inset shows how distance to nearest compacted trail was calculated by finding the centroid point for each segment within a given track and measuring the distance (m) to the nearest groomed snowmobile trail. Blue line denotes a snowmobile trail.

We randomly selected individual coyotes for backtracking using a computer generated randomization sequence (SAS Institute Inc., Cary North Carolina, USA) to avoid bias and ensure all coyotes were sampled randomly, yet equally. Once selected, coyotes were located by triangulation using ≥3 azimuths, and their position projected using LOCATE II (Nova Scotia Agricultural College, Truro, Nova Scotia, Canada). Once the track location was verified, a starting location for the actual track was then used to generate a starting location for the random track. Random tracks were created using digital layers from previously documented coyote tracks in a random direction and orientation, 3 km distance from the actual start point of the individual being tracked that day (Figure 1). This procedure and projection distance was used to ensure sampling independence (i.e., the actual and random tracks could not intersect) from the actual track and, for statistical purposes, for comparing data from the actual coyote track to random tracks [4].

The direction and projection of random tracks were generated randomly using SAS (SAS Institute Inc. 1999), by creating a randomized sequence selected from values between 1 and 360 (representing degrees); one randomization sequence was created for the direction, and one for the projection. Before going into the field, the random track created for that day was overlaid onto a topographic map using ArcGIS (ESRI, Redlands, California) to ensure field personnel were capable of conducting a track survey in the terrain where it had been randomly projected. If the random track had been projected in an avalanche path or dangerous/unattainable terrain, the track was re-projected to ensure safety of personnel, using a second set of projected numbers from the randomized sequence. If the terrain was considered acceptable, the random track layer was permanently saved onto a digital map, transferred to a handheld computer (Trimble GeoExplorer® series 3, Sunnyvale, California) and taken into the field. The only reason a track was ever re-projected was for safety reasons. Therefore ensuring random tracks were not projected in areas simply because they were easy to access or conduct track surveys in, eliminating potential surveyor bias of roads, terrain and snow compaction.

Backtracking began in the morning after night movements had taken place and before the snow column deteriorated. We conducted both actual and random track surveys by teams of 2 field personnel, taking measurements and recording data for ≥3 km of tracking. Start locations were reached using pre-existing trails to avoid additional compaction within the study area. Teams commenced backtracking of actual and random tracks simultaneously. Using the a handheld computer (Trimble GeoExplorer, Sunnyvale, California, USA), we collected all data in digital format using a datasheet generated with the computer software GPS Pathfinder Office (Trimble Navigation Limited, Westminster, Colorado, USA). At the start of each track, we recorded initial track information including observers, start time, start location, temperature, elevation, and a classification (high, medium, low) of snowmobile use in the area. Classes of high, medium, and low levels of snowmobile use were determined by visually assessing from the ground the amount of terrain covered by snowmobile tracks within a 1 km buffer of the track. A high classification was terrain with snowmobile tracks covering >60% of the ground within the buffer zone; snowmobile tracks covering <10% of the area was considered low; tracks covering 11–59% of the area was considered medium use.

During the actual backtrack of a coyote, Pathfinder software recorded UTM locations every 5 seconds along a given track. We recorded point locations every time a habitat change was encountered, organizing the track into distinct but consecutive segments [4]. We considered groomed trails a distinct habitat type. We documented coyote travel distance on and off snowmobile trails by track segments with start and end points marking transitions within habitats. We identified prey and predator track crossings as point locations, and recorded the number and species every time a prey or predator's track crossed a coyote travel path. During the entire backtrack (whether on or off a snowmobile trail), we measured the snow depth with every habitat change and every 200 m along the track using an avalanche probe (marked in cm) to measure from the snow surface to the ground. We documented an index of snow penetrability whenever the habitat changed and every 200 m along the entire backtrack by dropping a 100 g weight from 1 m above the snow surface and measuring the distance of penetration below the surface [4]. All established snowmobile trails, including groomed trails and off-trail snowmobile tracks, within 1 km of both actual and random tracks were recorded for measuring coyote distance to the nearest snowmobile trail. Trails made by field personnel while conducting the survey were not recorded as these occurred after the coyote had traveled the actual route the previous night. We measured all variables similarly along both actual and random tracks.

After the actual and random tracks were completed, data recorded on the Trimble units were downloaded and imported into GPS Pathfinder Office. Once imported, we differentially corrected the tracks to enhance location data quality. Tracks were then smoothed to eliminate bounce or GPS scatter caused by canopy cover or varying topography which can influence location accuracy [16]. All tracks were converted to ArcGIS files for analysis. We determined coyote travel distance to the nearest snowmobile trail (Figure 1) by calculating a centroid point for each segment along a given coyote track, then measuring the distance from the centroid point to the nearest snowmobile trail [4].

### Data and Statistical Analyses

We compiled backtrack data into track pairs by individual and date. We divided tracks into “compacted” and “non-compacted” categories, then divided into segments (based upon habitat transition) to compute mean prey track encounters (per km), mean predator track encounters (per km), mean snow depth (cm), and mean snow penetration (cm). Snow depth and penetration measurements were recorded every 200 m along both actual and random tracks. Once calculated for each segment, variables were averaged for compacted and non-compacted categories and the number of segments per track and mean segment distance were determined. We divided the distance traveled on and off snowmobile trails by the total track distance to determine percent use of snowmobile trails for each track pair.

To determine if coyotes traveled closer to a snowmobile trail during specific winter months, we compared distance from an actual coyote track to the closest snowmobile trail by month and year for both random and actual tracks. Our sampling unit was defined as each track pair, consisting of one actual and one random coyote track for any given day. Snow depth and snow penetration were averaged for each track segment to produce an overall average for each track. Distance from the actual coyote track to the nearest snowmobile trail was determined by calculating a distance for each segment on a given track and averaging those distances to produce a single mean distance for each track (Figure 1). Distances to the nearest snowmobile trail of actual tracks versus random tracks were compared using a paired sample t-test available in the ‘stats’ library using the t.test function with a paired sample specification (R software, version 2.6.2). This test calculates the difference between each actual and random paired tracks and then tests whether the average differs from zero.

To determine how snow depth and snow penetration encountered by coyotes influenced their use of snowmobile trails, we conducted correlation analyses by comparing the percentage of snowmobile trails used by coyotes during actual backtracks, versus the average snow depth encountered on snowmobile trails, the average snow depth encountered off trails, the average snow penetration encountered on trails, and the average snow penetration encountered off trails. We used linear regression analyses (SPSS 10.0, Chicago, Illinois, USA) to determine how each variable (snow depth on, snow depth off, snow penetration on, snow penetration off) influenced the percent use of snowmobile trails by coyotes.

To determine how large prey items influenced coyote movement, we compared the use of snowmobile trails on all actual tracks containing ungulate kills to those where ungulate kills were not documented. Tracks were categorized by either presence (1) or absence (0) of an ungulate kill, as documented during actual coyote backtracks. A distance ratio was calculated by dividing the actual distance traveled by a coyote (using snow-compacted surfaces) by the shortest possible travel distance possible, projected from start to finish points. This distance ratio was then compared between tracks with versus without an ungulate kill using a paired sample t-test available in the ‘stats’ library using the t.test function with a paired sample specification (R software, version 2.6.2) to determine whether coyotes preferentially used snowmobile trails when accessing large prey items rather than traveling the shortest direct distance.

The Multi-Response Permutation Procedure (MRPP) [17] was used to test for differences in variable means between random tracks and the actual tracks. We used the procedure ‘mrpp’ implemented in the R library ‘vegan’ (R software, version 2.6.2) [18]. MRPP tests whether there is a significant difference between 2 or more groups of sampling units, thus allowing us to compare variables from each track pair (actual and random) by day. This method is similar to a simple analysis of variance as it compares dissimilarities within and among groups based on *P*-value statistics [19]. The MRPP was applied to a number of variables; we calculated the means of each variable and assessed if they were significantly different between actual and random tracks. We first investigated differences in those means for the following variables: level of snowmobile use, snow depth, and snow penetration. To obtain a mean value of snowmobile use (classed as low, medium or high) for both actual and random tracks, we transformed snowmobile use into an ordinal variable (i.e., 1, 2, 3, replaced low, medium, and high). We also tested for differences in prey-related variables: rate of encountering tracks left by rodents, red squirrels, snowshoe hares, and ungulates. Additionally, we examined predator avoidance using the rate of wolf track encounters along the actual and random tracks.

We were interested in understanding which factors (i.e., coyote identity, level of snowmobile use, snow depth, snow penetration, rodents, red squirrels, snowshoe hares, ungulates, and wolf track encounters) on and off the snowmobile trails could explain the percentage of time coyotes spend on snowmobile trails (i.e., ‘%Track’). To address this question, we used beta-regression mixed models via the ‘betamix’ procedure implemented in the R library ‘betareg’ (R software version 2.6.2) [18]. Mixed beta regression models can be implemented in situations where the dependent variable (%Track) is continuous and restricted to the unit interval 0–1, such as proportions or rates [20]. Mixed beta-regression models can also accommodate repeated measurements nested within clusters; in our case,%Track measurements were nested within individuals whereby the response variable%Track was measured repeatedly for each individual. Accounting for an individual random effect of ‘coyote id’ will account for the nested nature of these repeated measurements within individuals. Because some of the covariates of interest had the potential to be collinear, we calculated a variance inflation factor (i.e., package ‘‘car,’’ procedure ‘vip’ in R version 2.6.0) [18] across covariates prior to model selection [21]. A variance inflation factor <5 indicated a lack of colinearity and ensured the covariates of interest could be simultaneously considered in the same regression. We first estimated a global model testing for additive effects of all of the covariates of interest [22]. We then removed covariates that did not have a significant effect on%Track (*P*>0.1). We repeated the process until each covariate had a significant effect on the response variable%Track (*P*≤0.1). Note that because model comparison of mixed models using information criteria such as AIC or BIC are still controversial (e.g., [23]), we decided to conduct model selection based on the significance of the explanatory variable only (i.e., P-values).

## Results

We captured and radio-collared 15 (4 F, 11 M) coyotes from August 2006 through February 2008. One individual was shot shortly after being radio-collared and 1 young coyote dispersed from the study area, leaving 13 individuals (4 F, 9 M) for sampling. We backtracked the 13 adult coyotes 57 times for a total of 265.05 km of actual coyote backtracking during 2 winters, 2006–2007 and 2007–2008. An additional 278.54 km of random tracks (n = 57 random tracks) were conducted during the same period. We averaged 4.62 backtrack pairs per animal (range  = 3–6, SD = 1.19); actual backtracks averaged a distance of 4.64 km in length (n = 57, range  = 1.56–12.21, SD = 1.69) with a mean of 34.10 track segments per backtrack (range  = 15–61, SD = 10.10). The random backtracks averaged a distance of 4.88 km in length (n = 57, range 3.14–11.81, SD = 2.50) with a mean of 25.68 track segments per backtrack (n = 57, range 1–39, SD = 9.82). Coyotes remained within any given habitat for a mean distance of 0.138 km during actual backtracks (range  = 0.001–1.149, SD = 0.120). During random backtracks, coyotes remained within any given habitat for a mean distance of 0.142 km (range  = 0.009–0.533). Actual backtracks were in areas most frequently categorized as medium snowmobile use areas (38.6%; 22 of 57 tracks) followed by low snowmobile use (35.1%; 20 of 57 tracks), and high snowmobile use (26.3%; 15 of 57 tracks). Random backtracks were in areas categorized as medium snowmobile use (38.6%), low snowmobile use (31.6%), and high snowmobile use (29.8%).

Coyotes used snowmobile trails for a portion of their track on all actual backtracks conducted (57 of 57 backtracks). For all actual backtracks combined, coyotes used snowmobile trails an average of 35% (range  = 0.02 – 86.68, SD = 23.02) of their travel distance. When traveling on trails, they traveled a mean continuous distance of 149 m per occurrence (range  = 0.1–352, SD = 0.90; Table 1), with a mean overall distance of 1.5 km spent on snowmobile trails per backtrack. Coyotes traveled on snowmobile trails during actual backtracks an average of 11.88 times per backtrack (range  = 1–33, SD = 6.28; Table 1). This was more than twice as often as during the random tracks (mean use of trails was 5.32 times on random tracks), and 3 times higher for the distance traveled on a trail than corresponding random tracks (mean continuous distance traveled on compacted snow per occurrence was 59 m on random tracks). Coyotes traveled significantly closer to snowmobile trails than random expectation (t = 13.67, df = 56, *P*<0.001), and selected shallower snow when traveling off trails (t = −3.909, df = 56, *P*<0.001).

**Table 1:** Comparisons of variable means (±SE) between compacted (used as a snowmobile trail) and non-compacted (undisturbed) track portions from actual (265.05 km) and random (278.54 km) coyote tracks recorded in the Togwotee Pass study area, northwestern Wyoming, 2006 – 2008.

	Actual tracks		Random tracks	
Variable	Compacted	Non-compacted	Compacted	Non-compacted
Total distance traveled (km)	85.94	179.58	34.07	244.47
Mean% distance of track	34.52±3.04	65.56±3.11	13.17±2.57	86.89±2.56
Mean snow depth (cm)	78.6±5.43	91.4±3.84	93.2±6.09	104.4±5.15
Mean penetration (cm)	11.9±0.98	19.3±1.11	12.9±1.43	20.2±1.01
# segments/track	11.9±0.83	21.89±1.35	5.32±0.66	20.37±0.97
Mean travel distance/segment (km)	0.124±0.01	0.105±0.01	0.078±0.01	0.206±0.01
Distance to snowmobile trail (m)	0	142.5±27.91	0	238.6±34.82
Predator track crossings	5.38±0.79	3.61±0.83	6.30±0.50	4.87±0.43
Wolves/km	0.53±0.24	0.19±0.11	0.11±0.09	0.19±0.16
Prey track crossings	12.74±1.45	12.18±1.53	5.31±1.06	16.56±1.60
Rodents/km	0.68±0.27	0.27±0.06	0.85±0.43	0.49±0.14
Red squirrels/km	2.60±0.66	3.10±0.51	1.54±0.59	3.22±0.43
Snowshoe hares/km	4.78±1.14	6.54±0.99	12.66±9.45	5.73±1.24
Ungulates/km	1.65±0.85	2.26±0.87	0.15±0.14	0.72±0.22

When averaged by track, coyotes crossed more predator tracks on actual tracks than on random tracks (actual: mean  = 5.82/km [range  = 0–34.85, SD = 6.31]; random: mean  = 3.09/km [range = 0–22.6, SD = 3.82]; t = 6.552, df = 56, *P*<0.001). Although more tracks of prey were encountered on actual backtracks than on random tracks (actual: 11.27/km, range  = 0–54.75, SD = 11.60; random: 9.96/km, range  = 0–67.49, SD = 12.13), this effect was not significant (*P*>0.30) when analyzed by track. Wolf tracks were crossed at similar rates (*P*>0.40) on both actual (mean  = 0.35/km, range  = 0–7.69, SD = 1.26) and random tracks (mean  = 0.37/km, range = 0–9.36, SD = 1.52). Snowshoe hares (SSH) were the predominant prey track crossed on both actual and random tracks, with encounter rates as high as 24.26 SSH/km on actual tracks (mean  = 5.83, range = 0–24.26, SD = 6.42) and 56.94 SSH/km on random tracks (mean  = 5.77, range = 0–56.94, SD = 9.85) but was not significantly different (*P*>0.20) between actual and random tracks. Grouse were encountered more on actual tracks than on random tracks (t = 0.063, df = 56, *P* = 0.063).

**Figure 2:**
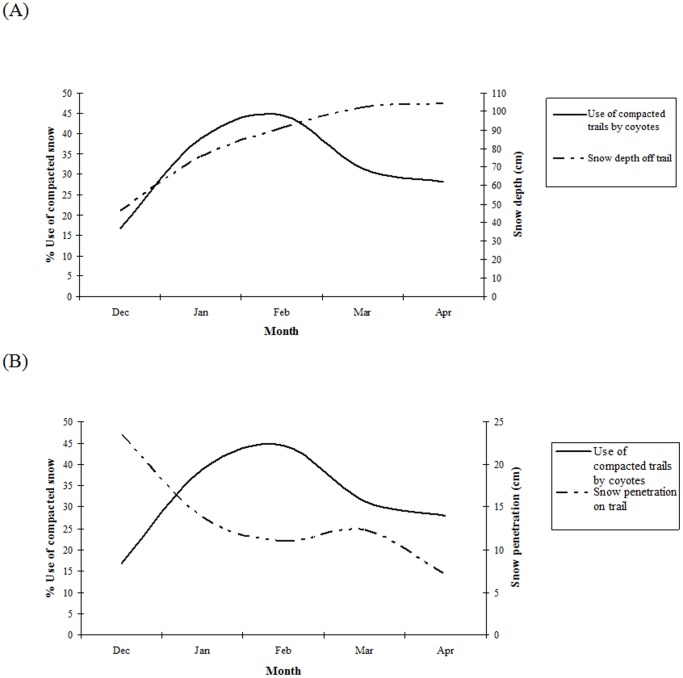
Percent use of snowmobile trails by coyotes in relation to (A) snow depth off the snowmobile trail, and (B) snow penetrability on the snowmobile trail, for each winter month, December 2007 through April 2008, northwestern Wyoming.

We observed an inverse relationship between the overall percent that coyotes used snowmobile trails and snow penetration when plotted by month (Figure 2). When we compared the effects of snow condition (snow depth on trail and off trail, as well as snow penetration on trail and off trail; 4 variables total) on the percentage of snowmobile trails used by coyotes by day, the relationship was significant. However, only 20.3% of the variation in use of snowmobile trails was explained by both snow depth and snow penetration (*F* = 3.31, df = 2, *P* = 0.017; see also Table 2). Regardless, coyotes increased their use of snowmobile trails as snow penetration off the snowmobile trails increased (became less supportive) and as snow depth increased (Figure 2), and as snow penetration on the snowmobile trails decreased (became more supportive). Additionally, coyotes increased their use of snowmobile trails as snow depth both on and off snowmobile trails increased.

**Table 2:** Linear regression analysis testing for the effects of snow depth on snowmobile trails, snow penetration on snowmobile trails, snow depth off snowmobile trails, and snow penetration off snowmobile trails on the percent distance coyotes use a snowmobile trail.

Variables	*β* estimates	Std. error	t-statistic	*P*
Snow depth (on trail)	0.396	0.124	3.197	0.002
Snow penetration (on trail)	−1.357	0.492	−2.758	0.008
Snow depth (off trail)	−0.405	0.169	−2.393	0.020
Snow penetration (off trail)	0.831	0.413	2.011	0.050

= 265 km) surveyed in the Togwotee Pass study area, northwestern Wyoming, 2007–2008. This analysis utilized all actual tracks (total distance

When comparing ratios between the mean distances of the shortest possible travel route and the actual travel route chosen by coyotes where ungulate kills were present, we found a significant difference in the amount of use on snowmobile trails (*P*<0.0001). The distance ratio was significantly higher in cases where there was an ungulate carcass (mean  = 5.25, range  = 3.62 – 6.25), compared to a situation where there was no ungulate carcass (mean  = 3.08, range  = 2.54 – 4.25), suggesting preferential use of snowmobile trails by coyotes to access ungulate carcasses. Coyotes preferred to meander along a snowmobile trail leading to a carcass rather than travel a more direct, but off trail, route of travel.

All variables were significant, with the exception of the mean level of snowmobile use and wolf track encounter rate, between random and actual tracks (Table 3). These non-significant results suggested first that snowmobile use did not explain coyote backtracks more than random expectation; it also suggested that the presence of wolves did not explain coyote track use more than randomly expected. Snow depth and snow penetration variables on the other hand indicated coyotes preferentially used shallower tracks where snow penetration and snow depth were lower than random expectation (Table 3). Coyotes preferentially used tracks where red squirrel track encounters were higher than random expectation, but where rodent and snowshoe hare track encounters were lower than randomly expected (Table 3).

**Table 3:** Multi-Response Permutation Procedure (MRPP) testing for differences in variable means (±SE) between actual tracks (265 km) and random tracks (279 km) in northwestern Wyoming, 2007–2008.

Variables	Actual track	Random track	*P*
Snowmobile use^a^ Recreational use	20(L)/ 22(M)/ 15(H)	14(L)/ 27(M)/ 16(H)	0.801
Snow depth (cm)	85.02±3.36	99.26±3.94	0.005
Snow penetration(cm) (cm)	15.59±0.82	17.23±0.91	<0.001
Rodents/km	0.47±0.14	0.57±0.15	0.004
Red squirrels/km	2.85±0.42	2.68±0.36	<0.001
Snowshoe hares/km	5.66±0.75	10.37±5.04	0.012
Ungulates/km	1.96±0.60	0.49±0.14	0.077
Wolves/per km	0.36±0.21	0.17±0.12	0.379

=  low, M  =  medium, H  =  high.^a^ Snowmobile use: L

Because all variance inflation factors were <5, all variables used in the beta regression mixed models did not show any colinearity issues [21]. Beta regression models indicated coyotes were exploiting snow-compacted routes, with their use directly related to the amount of snow compaction available. The best performing model retained an effect of snowmobile use (i.e., low, medium, or high) whereby snowmobile use had a progressive negative effect on%Track (Table 4; high use: *β* = −0.0421; *P* = 0.8544; medium use: *β* = −0.8988; *P*<0.001; low use: *β* = −1.1308; *P*<0.001). However, only lower (*P*<0.001) and medium (*P*<0.001) levels had a significant negative effect on%Track (Table 4). The best performing model also retained an effect of rodent track crossings on snowmobile trails on the time spent by coyotes on snowmobile tracks ‘%Track’ (Table 4). The abundance of rodent tracks encountered on the snowmobile trails positively and significantly influenced the percentage of time a coyote spent on snowmobile trails (*β* = 0.1411; *P* = 0.0407).

**Table 4:** Results pertaining to the best performing beta regression mixed models for the effects of various covariates of interest (e.g., snowmobile use, rodent track encounters) on the amount of time coyotes spend on snowmobile trails (i.e.,%Track), northwestern, Wyoming, 2007–2008.

Explanatory variables	*β*	SE	z-test	*P*
Snowmobile use (low)	−1.1308	0.2146	−5.2696	<0.001
Snowmobile use (medium)	−0.8988	0.2000	−4.4947	<0.001
Snowmobile use (high)	−0.0421	0.2297	−0.1835	0.8544
Rodent encounters/on tracks	0.1411	0.0690	2.0462	0.0407

## Discussion

Our findings showed that coyote use of snowmobile trails was associated with presence of a food source (i.e., an ungulate carcass) demonstrating their ability to preferentially use trails to facilitate access, and coyote use of snowmobile trails was related to the availability of trails. Overall, coyote use of snowmobile trails was related to both snow compaction and snow depth; as snow depth and penetrability off trails increased, coyote use of snowmobile trails increased (Figure 2). Essentially, the snow column characteristics were most influential on how much time coyotes spent on snowmobile trails. In the early months of winter, snow depth was low, yet the snow column remained dry and the coyotes easily traveled through the study area. As winter progressed and snow depth increased and snow penetrability (i.e., opposite of supportiveness) increased, coyotes spent more travel distance on snowmobile trails. As spring approached, the snow depth remained high but penetrability decreased (i.e., became more supportive) and hence the coyotes traveled less on snowmobile trails because the snow column off trail was more supportive.

**Figure 3:**
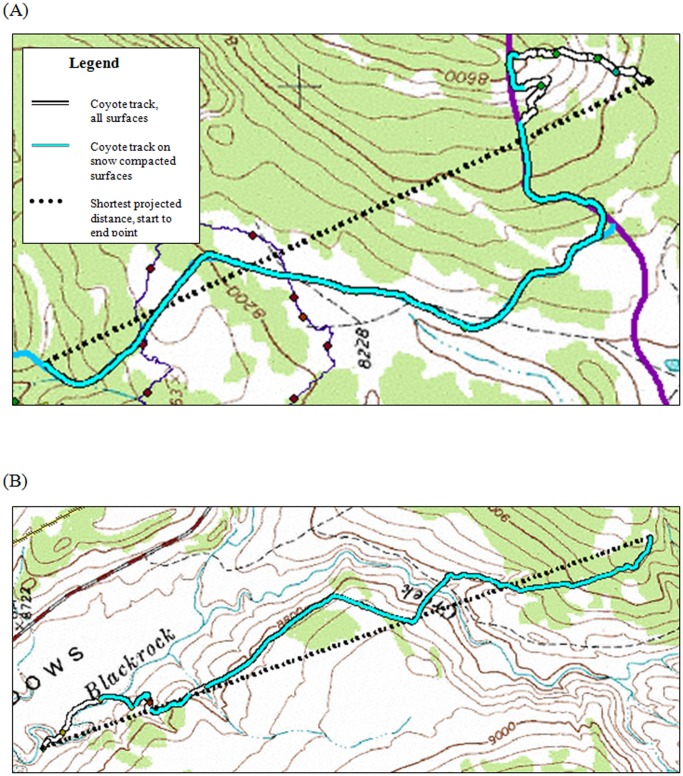
Examples of coyote travel paths in the presence of snowmobile trails: (A) Male coyote 05 on 4 January2008, and (B) Male coyote 15 on 3 April 2008, northwestern Wyoming, 2007–2008.

We documented coyote use of snowmobile trails on every backtrack, suggesting that even though coyotes are only using snowmobile trails an average of 34.5% of their overall track distance, there was a strong association between coyotes and snowmobile trails in our study area. Analysis of percent coyote use of snowmobile trails and snow depth by month, showed coyotes preferentially used trails during core winter months (January through March; Figure 2). Use of trails was less during December and April, when temperatures were higher, and the snow was wetter and more compacted due to melting and freezing cycles. During these months, conditions were more similar to those typical of many areas where lynx and coyotes coexist [4]. Based on results from Kolbe [4], they were not able to conclude that “compacted snowmobile trails facilitated coyote movements” in their study area. We suggest snow conditions in northwestern Wyoming are much drier and less supportive than those documented in western Montana. Unlike Kolbe's findings, there were several instances when coyotes used snowmobile trails almost exclusively over the course of a 3 km backtrack (Figure 3).

Extensive use of compacted trails was not the only finding contradictory to those of Kolbe [4]. In addition to coyotes using snowmobile trails more than expected, we also found the mean distance coyotes traveled away from snowmobile trails was shorter on actual versus random tracks. While Kolbe [4] suggested coyotes can behaviorally adapt by selecting shallower and more supportive snow to travel, hunt, and utilize resources, rather than rely on snowmobile compacted surfaces, we suggest the level of behavioral adaptation needed to persist in such habitats is dictated by the snow characteristics in the area. Therefore, adaptations, behaviors and use of compacted surfaces will differ based on geographical location and ultimately, characteristics of the snow column.

Coyotes crossed more prey (ungulates and squirrels) tracks and fewer predator tracks during actual backtracks while traveling on compacted snow than on random backtracks. Ungulates and red squirrels were the only prey species showing a higher than expected track crossing rate on actual compacted versus random compacted coyote backtracks, suggesting selection of snowmobile trails may be associated with those species rather than with other prey. Based on winter diet analyses [24], coyotes may be selecting travel paths based on ungulate presence. Although coyote predation on ungulates has been reported [25]–[26], killing of ungulates by coyotes is considered risky due to the possibility of injury and low success rates [25]–[27]. Therefore, the associations between coyote travel paths and ungulate presence was not likely due to direct killing by coyotes, rather this association could be exploiting kills made by other predators; evidence indicated most ungulate carcasses encountered were wolf kills scavenged by coyotes. Scavenging of wolf kills can be advantageous to coyotes, provided they can exploit the kill while minimizing costs of gaining access and managing the risk posed by wolves [28].

During several backtracks, coyotes used snowmobile trails to travel from one forested cluster to another where snow was shallower under trees and behaviors such as chasing, digging or hunting rodents occurred. This could possibly provide an explanation for the association between coyote travel paths and red squirrel encounters. The association with red squirrel track crossings on actual compacted coyote backtracks could be explained if coyotes were selecting areas having a high occurrence of red squirrels because of their association with squirrel middens. When backtracking coyotes, we found several instances where coyotes were digging in squirrel middens, and diet analyses [24] showed coyotes were not targeting red squirrels themselves, but raiding middens (i.e., caches of pine nuts).

Coyotes may be more adaptable and tolerant of disturbance caused by snowmobiles than other predators. Snowmobile trails are used frequently by people and constantly managed for daily use which may be a deterrent to less tolerant wildlife species. Coyotes, however, may adapt to these human-modified areas and use them to their advantage for traveling, hunting, and accessing desirable habitat patches. Another plausible explanation for the high use of managed snowmobile trails by coyotes is the association of movement patterns and the use of roads because of its structure. Coyotes in Seeley Lake, Montana, may have selected for road structure and location rather than the snow conditions on them [4]. While road structure [4] is a plausible explanation in regions where snow conditions result in more supportive or unaltered travel conditions, it was not a likely explanation for our study area because coyote travel patterns changed based on snow conditions (depth and supportiveness; Figure 2), and coyotes in our area traveled closer to snowmobile trails than random expectation. We believe this behavior was a direct result of facilitated travel on compacted surfaces, several of which coincidentally were managed for winter recreation.

Energetic trade-offs become important in winter when harsh conditions carry high energetic costs and survival requires a balance of nutritional intake with energy expenditure. Predators must either change their behavioral patterns to utilize resources in deep snow habitats, or shift their range to an area where food is more accessible and acquisition of resources less energetically expensive. While coyotes have been shown to shift territory use to lower elevations during the winter [29], this was not documented in our study. Instead, our findings were similar to another study [4] which documented little change in the mean elevation of coyote backtracks during winter. Based on year-round monitoring of individuals using telemetry, we were able to determine that coyotes resided in their home ranges throughout the year and did not demonstrate seasonal shifts due to deep snow.

Our study provided insight on the relationships between snowmobile trails and their influence on coyote movements in the southern periphery of lynx range. While direct impacts of snowmobiles on lynx were not documented, the potential impacts of a main competitor, the coyote, are worth mentioning. Due to their use of snowmobile trails, coyotes have the potential to access areas of habitat that might normally be too energetically difficult to access in deep snow. Lynx, with their superior body mass to foot load, can access habitats containing deep snow that coyotes might typically avoid. In addition, expansion of current winter recreation use areas may create persistent travel corridors that could be utilized by coyotes. Since coyote use of snowmobile trails was related to how much was available, coyote movements could possibly be altered by limiting snow compaction. Bunnell [3] suggested the use of snowmobiles may result in consistent compacted trails within lynx conservation areas which may be detrimental to local lynx populations in the Intermountain West. Furthermore, they suggested minimizing or rotating compaction areas (thereby limiting potential impacts by coyotes) as a strategy for management agencies concerned with protecting habitats needed to sustain lynx and their prey. Our findings support this management strategy, but further research should be conducted to determine whether the suggestion of Bunnell [3] is practical and could be implemented successfully in areas where lynx conservation is a concern.
